# Bullous Pemphigoid Secondary to Dipeptidyl Peptidase-4 (DPP4) Inhibitor: A Case Report

**DOI:** 10.7759/cureus.88054

**Published:** 2025-07-16

**Authors:** Raquel Rosa, Marta Sousa Cardoso, Inês Pinheiro Silva, Joana Leal, Bárbara Torres

**Affiliations:** 1 USF São Domingos, ULS Lezíria, Santarém, PRT; 2 USF Côrtes d'Almeirim, ULS Lezíria, Santarém, PRT

**Keywords:** adverse reaction, bullous pemphigoid, dpp4i, geriatrics, skin eruption

## Abstract

Bullous pemphigoid (BP) is a rare acquired subepidermal autoimmune disease characterized by the presence of blisters on the skin. Recently, a possible association has been reported between the use of DPP-4 inhibitors, an oral antidiabetic, and an increased risk of BP. The mechanisms underlying this increased risk remain poorly understood. We present the case of an 80-year-old diabetic man who developed scattered blistering pruritic lesions on the trunk and limbs, which had been progressively worsening over approximately 6 months and difficult to resolve. After investigating his medical history and observing significant improvement following the administration of topical corticosteroids, the clinical presentation was associated with the use of a DPP-4 inhibitor. In conclusion, the case presented suggests a cause-and-effect relationship between BP and DPP-4 inhibitors. Early recognition of these adverse reactions is crucial to establish appropriate interventions to prevent the progression of conditions such as BP.

## Introduction

Bullous pemphigoid (BP) is a rare, acquired subepidermal autoimmune disorder characterized by the presence of blisters on the skin. It is associated with the presence of autoantibodies primarily directed against two hemidesmosomal proteins: BP180 (also known as BPAg2 or type XVII collagen) and BP230 (also known as BPAG1e - epithelial isoform), which are components of the junctional adhesion complexes responsible for dermo-epidermal cohesion [1,2]. When binding occurs between the antibody and antigen, inflammatory mechanisms are activated, including mast cell, neutrophil, and eosinophil activation with the release of inflammatory cytokines and proteolytic enzymes, resulting in dermo-epidermal junction damage [1,2].

It has an estimated incidence of 6 to 43 new cases per million people per year worldwide. BP accounts for approximately 80% of immune-mediated blistering diseases and reaches a peak incidence between 60 and 80 years of age [1,2]. Studies show that in recent years, the incidence of BP has increased, which may be due to greater awareness among clinicians regarding blistering lesions, an increase in patients with conditions associated with BP (such as dementia and disabling neurological diseases), and increased use of medications that may be linked to BP [1].

The clinical presentation of BP includes a prodromal phase with moderate to severe pruritus that may occur alone or in association with maculopapular eczematous or urticarial lesions. This phase may last for weeks to months before the appearance of blisters [2]. About 20% of patients do not progress to the blistering phase [3]. BP lesions appear as vesicles or blisters on erythematous or normal skin, are tense in consistency, and range from 1 to 4 cm in diameter. Hemorrhagic lesions may also be present. These lesions last for several days before rupturing and forming erosions and crusts [2]. BP lesions are distributed on the limbs, trunk, and abdomen.

Diagnosis is clinical and relies on recognizing the typical clinical and epidemiological features of BP. It may be confirmed by skin biopsy, histological evaluation, and immunohistochemistry [2].

Treatment involves the use of topical and/or systemic immunomodulatory therapy depending on disease severity and patient comorbidities. In cases of localized disease affecting <20% of the body surface in elderly patients, high-potency topical corticosteroids may be considered. In more severe cases with extensive disease, systemic corticosteroid therapy should be considered, such as prednisolone at a dose of 0.5 to 1 mg/kg per day for two weeks, followed by slow tapering over 6 to 9 months [2,4]. If symptoms persist, other immunosuppressive agents may be used, such as azathioprine, mycophenolate mofetil, methotrexate, and cyclosporine. As a last resort, in resistant cases, intravenous immunoglobulin (IVIG), rituximab, or omalizumab may be alternatives [2]. BP can have a chronic course, making long-term assessment of these patients essential, as outcomes may vary.

Possible complications of BP include bacterial infection of the lesions, increased susceptibility to herpes simplex and varicella-zoster infections, and adverse effects from the instituted treatment [2].

One of the main triggers for the development of autoantibodies responsible for BP is systemic medication, with several drug classes being implicated, including diuretics, non-steroidal anti-inflammatory drugs, amoxicillin, PD-1/PD-L1 inhibitors, DPP-4 inhibitors, and TNFα inhibitors [1,2,5].

The possible association between the use of dipeptidyl peptidase-4 (DPP-4) inhibitors - drugs approved for the treatment of type 2 Diabetes Mellitus - and an increased risk of developing BP has recently been reported in several studies [2,5,6,7]. DPP-4 inhibitors prevent the degradation of glucagon-like peptide 1 (GLP-1). DPP-4 is associated with anti-inflammatory effects in autoimmune diseases and arthritis [5]. The mechanisms explaining this increased risk of BP associated with DPP-4 inhibitors remain poorly understood. One hypothesis suggests these drugs may trigger the production of antibodies directed against the basement membrane or structurally similar antigens, causing subepidermal blister formation. Additionally, some research indicates that inhibition of DPP-4 may promote eosinophil recruitment to the dermis, potentially contributing to blister formation and tissue damage in BP patients [5].

According to several clinical studies and meta-analyses, BP development is mainly associated with the molecule linagliptin and, to a lesser extent, vildagliptin [5,6].

This clinical case aims to raise awareness about the development of this condition, which, although quite rare, leads to reduced patient quality of life and may be associated with medications frequently used in Family Medicine practice.

## Case presentation

An 80-year-old male presents with a history of type 2 diabetes mellitus, dyslipidemia, ischemic heart disease (acute myocardial infarction in 2017 with four coronary stents placed), diverticulosis, benign prostatic hyperplasia, and recurrent basal cell carcinoma (monitored in private dermatology consultations). His regular medications include metformin 500 mg twice daily, gliclazide 30 mg, linagliptin 5 mg, clopidogrel 75 mg, bisoprolol 2.5 mg, ramipril 2.5 mg, atorvastatin 20 mg, tamsulosin 0.4 mg, and finasteride 1 mg.

During a diabetes follow-up consultation with his family doctor, the patient reports the emergence of pruritic erythematous papular lesions on the trunk and upper limbs, progressing over six months. He also describes generalized pruritus, particularly affecting his upper limbs and hands, which does not improve with skin hydration. Some lesions evolve into blisters that rupture and form crusts. He denies any correlation between the onset of lesions and changes in hygiene products, exposure to new substances, or dietary alterations. He has attempted more frequent application of moisturizer without success. On his own initiative, he applied a topical corticosteroid (methylprednisolone aceponate) purchased over the counter, which he found to be the only intervention providing relief.

**Figure 1:**
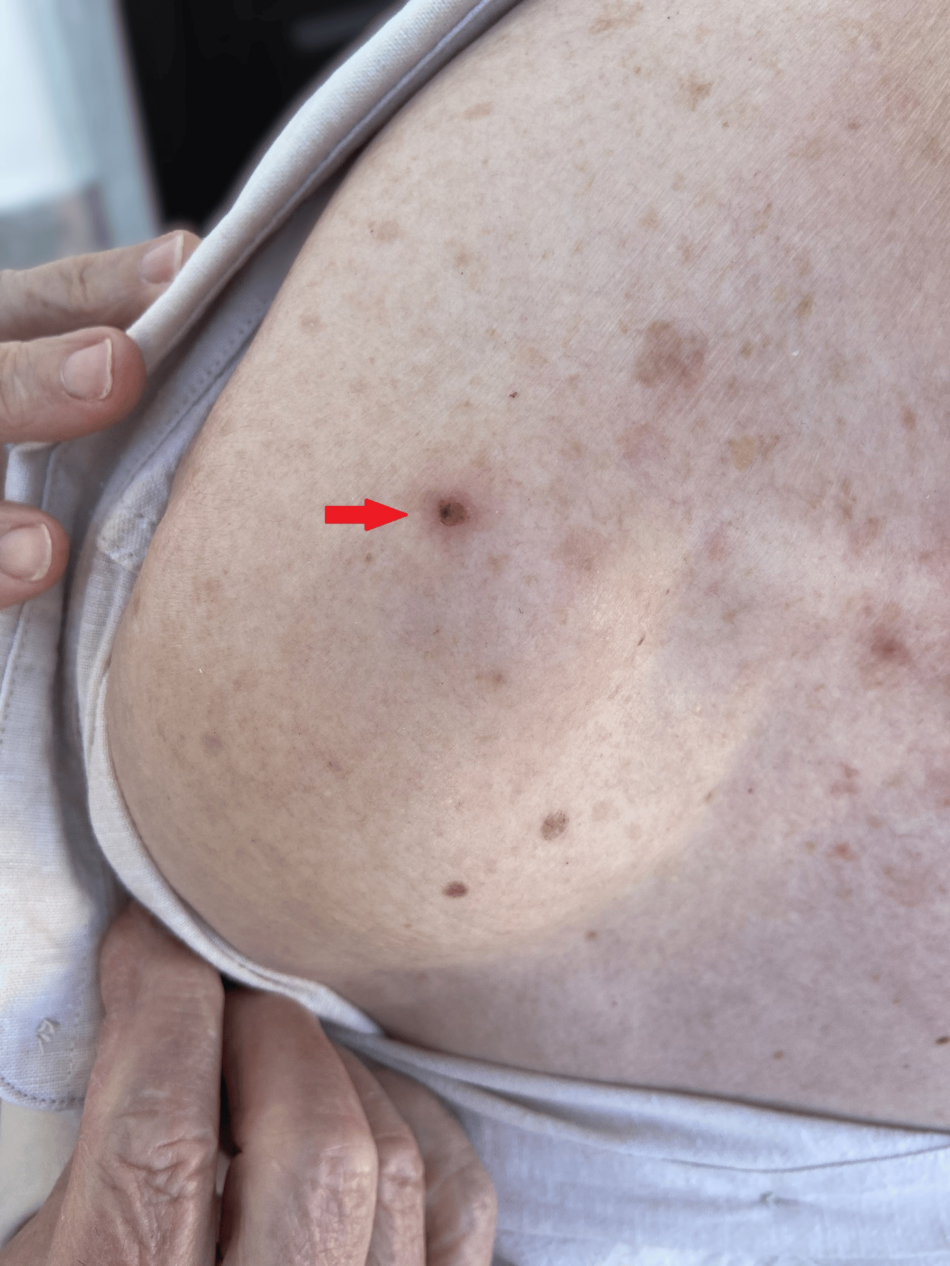
Crusted lesion in the upper arm

**Figure 2:**
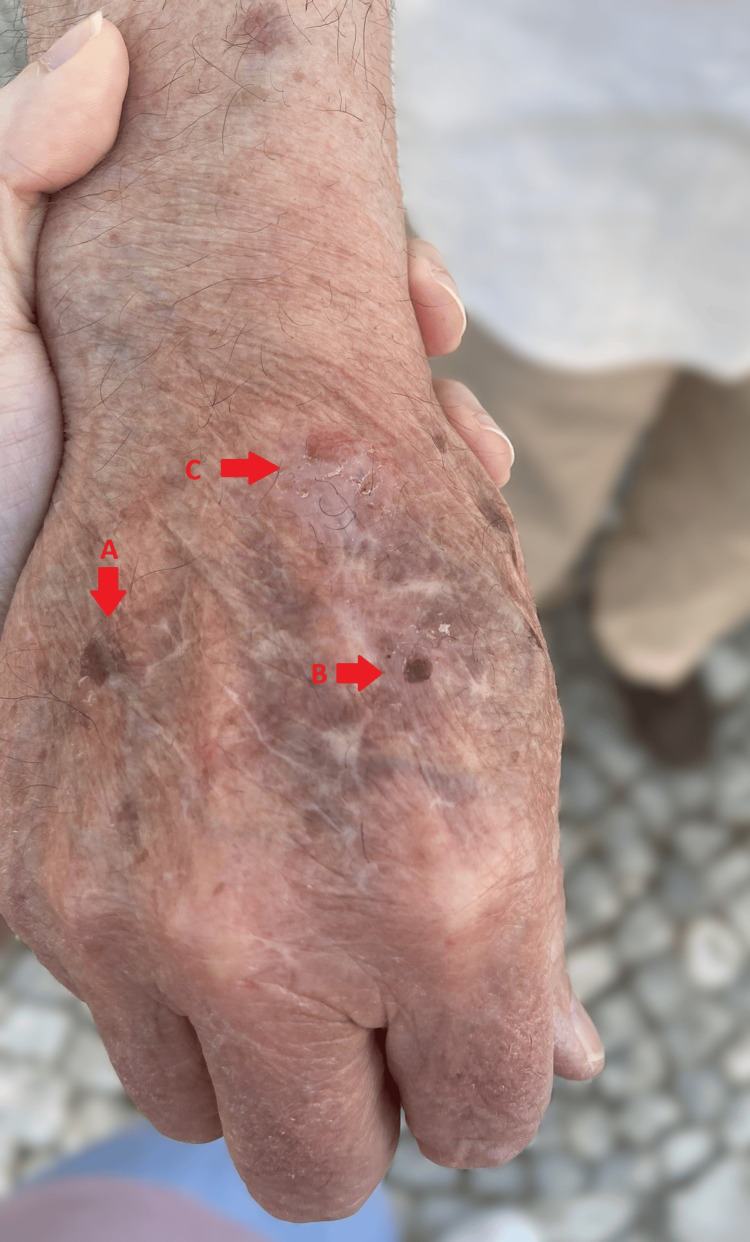
Crusted (A), blistered (B), and healed (C) lesions in the hand

**Figure 3:**
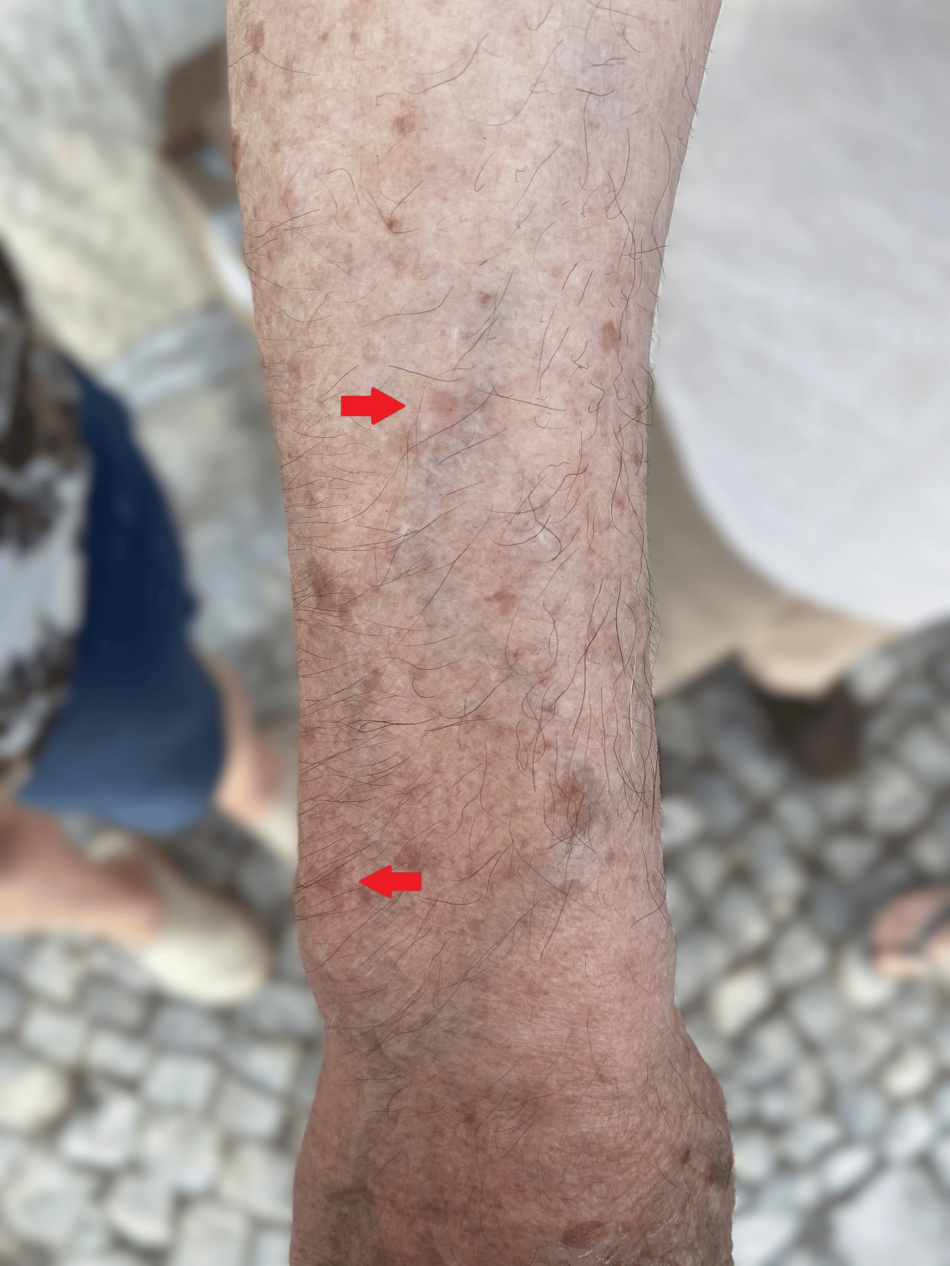
Initial fase lesions (erythematous plaques)

On physical examination, multiple small papular lesions, some blistering and others crusted, are observed on an erythematous base over the upper trunk and limbs. Signs of scratching are evident, particularly on the upper limbs (Figures 1-3). No indications of severe xerosis are noted.

A review of clinical records reveals that symptoms began approximately two months after an adjustment in diabetes medication. Due to poor metabolic control with metformin and gliclazide alone (intolerance to SGLT2 inhibitors due to recurrent balanitis), linagliptin, a DPP4 inhibitor, was introduced.

Given the characteristics of the lesions, BP was suspected. The temporal association with linagliptin initiation raised concerns, leading to the drug's discontinuation and an urgent dermatology referral. The patient opted to consult his private dermatologist, who confirmed the clinical diagnosis of BP and contraindicated further use of DPP-4 inhibitors. As the condition was mild, topical corticosteroid treatment was initiated for two weeks, with follow-up evaluation. The lesions improved substantially following treatment and drug withdrawal.

Four months later, the patient underwent systemic corticosteroid therapy for newly diagnosed ulcerative colitis, during which BP lesions fully resolved. After corticosteroid tapering and cessation, BP lesions recurred, though more mildly, and are now managed with occasional topical corticosteroid application.

## Discussion

BP is a rare autoimmune skin condition that typically affects elderly patients, often with multiple comorbidities [1,2]. Its lesions are vesicles or tense blisters that lead to erosions and crusting. These lesions are frequently preceded by moderate to intense generalized pruritus, which may or may not be associated with maculopapular eczematous or urticarial lesions [1,2].

It is known that BP can be triggered by systemic medications being used by the patient [1,2,5,6,7]. Several pharmacological classes are involved, but one of the most commonly used in clinical practice is DPP-4 inhibitors, prescribed for the management of type 2 Diabetes Mellitus [5,6,7].

This clinical condition, due to its characteristics, significantly compromises the patient’s quality of life. A high level of clinical suspicion is required for diagnosis, as it is uncommon to encounter patients with this condition, and the clinical presentation is not always straightforward or textbook [1,2,5].

In the presented case, the patient had a mild form of the disease, with a presumptive diagnosis based on clinical features and suspicion due to the recent initiation of linagliptin (a DPP-4 inhibitor) [1,2,5]. Despite the mild clinical presentation, the discomfort was marked, and the prodromal phase was intense. No confirmation tests (for example, skin biopsy) were done, as the clinical features and therapeutic response strongly supported the diagnosis of BP.

Discontinuation of the medication in question was essential to controlling disease progression. As this was a mild case, the blistering lesions were managed with topical corticosteroids [2]. However, this is not always possible, and in more severe cases, systemic corticosteroid therapy may be necessary [2]. It was also notable that near-complete resolution of the lesions occurred with immunosuppressive medication being used by the patient for another medical condition, further supporting the diagnosis [2].

The prognosis of BP is uncertain; it is a chronic disease that may require long-term treatment. Symptoms may persist for several months to years before fully resolving [2,4].

## Conclusions

BP is a rare autoimmune disease, but one that can significantly impact patients’ quality of life. Despite its rarity, it is crucial for physicians to remain vigilant regarding the signs and symptoms of this condition, particularly given its potential association with commonly prescribed medications for highly prevalent diseases - such as type 2 diabetes mellitus - and oral antidiabetic agents like DPP-4 inhibitors, whose link to BP is well established.

This case highlights the importance of that association and underscores the need to be alert to it so that the offending drug can be discontinued early and specific treatment initiated as soon as possible. Such timely intervention helps prevent progression to more severe stages of the disease, which carry greater disability.

Therefore, early recognition of the connection between BP and possible drugs in the patient’s usual medication regimen is essential to halt disease progression and allow for the prompt withdrawal of implicated therapies.
